# The complete chloroplast genome of *Gentiana macrophylla*

**DOI:** 10.1080/23802359.2017.1347831

**Published:** 2017-07-11

**Authors:** Xiaofan Wang, Na Yang, Jiao Su, Hui Zhang, Xiaoyan Cao

**Affiliations:** Key Laboratory of the Ministry of Education for Medicinal Resources and Natural Pharmaceutical Chemistry, National Engineering Laboratory for Resource Development of Endangered Crude Drugs in Northwest of China, Shaanxi Normal University, Xi'an, China

**Keywords:** Chloroplast genome, *Gentiana macrophylla*, cpSSRs, phylogeny

## Abstract

*Gentiana macrophylla* is a perennial medicinal plant in the family Gentianaceae. Natural sources for *G. macrophylla* plants are being exhausted due to over-collection and need urgent conservation. The complete chloroplast DNA sequence of *G. macrophylla* (GenBank accession number: KY856959) was determined in our study. The size of chloroplast genome of *G. macrophylla* is 149,916 bp, including a large single-copy (LSC) region of 82,911 bp and a small single-copy (SSC) region of 17,095 bp separated by a pair of identical inverted repeat regions (IRs) of 24,955 bp each. A total of 130 genes were successfully annotated containing 86 protein-coding genes, 36 tRNA genes and 8 rRNA genes. The GC content is 37.7%. 33 chloroplast simple sequence repeats (cpSSRs) of *Gentiana macrophylla* were detected, in which 32 of them are mononucleotide repeats. Phylogenomic analysis strongly supported the close relationship of *G. macrophylla* and other 5 species in Gentiana.

## Introduction

*Gentiana macrophylla* is a perennial medicinal plant in the family Gentianaceae. Its dried roots have been widely used for more than 2000 years as ‘Qinjiao,’ a traditional Chinese herbal medicine (Hua et al. 2014). Natural sources for *G. macrophylla* plants are being exhausted due to over-collection (Cao et al. 2005; Yin et al. 2009). Chloroplast genomes typically present a conserved quadripartite structure composed of a large single-copy (LSC) region and a small single-copy (SSC) region, which are separated by two copies of inverted repeats (IRs) (Jansen et al. 2005). Chloroplast DNA (cpDNA) has been used broadly as a universal method in research on phylogenetic resolution of plant evolutionary and biodiversity, because of its simple structure, highly conserved sequence and maternal inheritance (Tian & Li 2002). In the present study, we reported the complete chloroplast genome sequence of *G. macrophylla* based on the next-generation sequencing method and provide essential resource for both conservation and utilization of this important species. The annotated cpDNA has been deposited into GenBank with the accession number KY856959.

Fresh leaves of *G. macrophylla* were collected from greenhouse of National Engineering laboratory for Resource Developing of Endangered Chinese Crude Drugs in Northwest of China (108°53′30″E, 34°9′14″N; NSII accession number 13475, http://www.nsii.org.cn/2017/specimen.php?id=2393479) in Xi′an city. Those leaves were stored in the refrigerator at −80 °C. Pure cpDNA that had checked was sequenced using the Illumina HiSeq 2500 platform in Biomarker Technologies in Beijing, with paired-end 125 bp sequencing method. The complete chloroplast genome was assembled and annotation using CLC Genomics Workbench (CLC Bio, Aarhus, Denmark) and Geneious R9 (v9.0.2) coupled with manual corrections start and stop codons. *Gentiana crassicaulis* was used as reference for assembled and annotation. The size of chloroplast genome of *G. macrophylla* is 149,916 bp, including a large single-copy (LSC) region of 82,911 bp and a small single-copy (SSC) region of 17,095 bp separated by a pair identical inverted repeat regions (IRs) of 24,955 bp each. A total of 130 genes were successfully annotated containing 86 protein-coding genes, 36 tRNA genes and 8 rRNA genes, which is similar to reported *Gentiana lawrencei var. farreri* chloroplast genome (Fu et al. 2016). Most genes occur as a single copy, except for 16 of them with double copies within the IR regions. Fifteen genes contained one intron, while three genes had two introns. The GC content is 37.7%.

Thirty-three chloroplast simple sequence repeats (cpSSRs) of *G. macrophylla* were detected with minimal repeat numbers of 10, 6, 5, 5, 5 and 5 for mono-, di-, tri-, tetra-, penta- and hexa-nucleotides, respectively. There were 32, 1 mononucleotide, dinucleotide repeats, respectively, and no other SSRs were detected.

**Figure 1.**
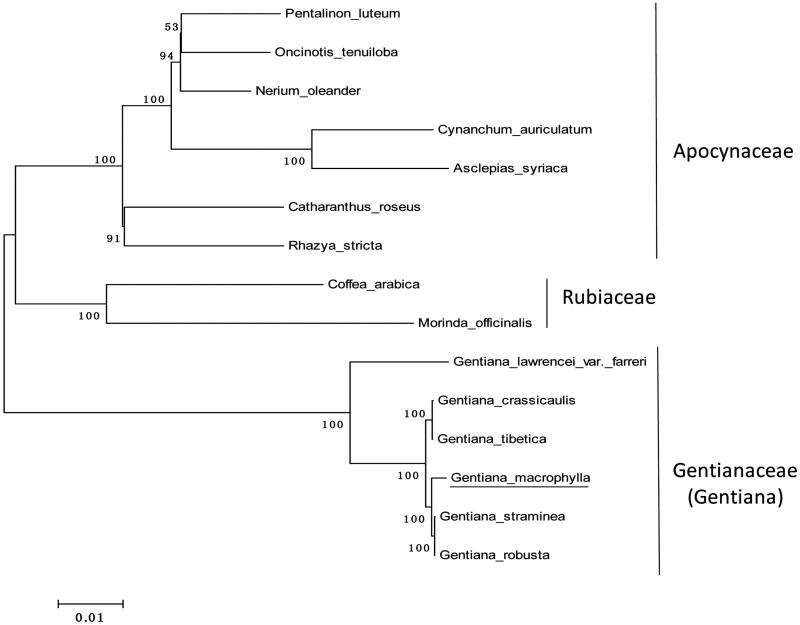
Phylogeny of 15 species within the Gentianales based on the neighbour-joining (NJ) analysis of the complete chloroplast genome sequence. The gene’s accession number for tree construction is listed as follows: *Gentiana straminea* (NC_027441); *Gentiana crassicaulis* (NC_027442); *Gentiana tibetica* (NC_030319.1); *Gentiana robusta* (KT159969.1); *Gentiana lawrencei var. farreri* (KX096882.1); *Catharanthus roseus* (NC_021423); *Rhazya stricta* (NC_024292); *Nerium oleander* (NC_025656); *Pentalinon luteum* (NC_025658); *Oncinotis tenuiloba* (NC_025657); *Cynanchum auriculatum* (NC_029460); *Asclepias syriaca* (NC_022432); *Coffea arabica* (NC_008535); and *Morinda officinalis* (NC_028009).

To ascertain its phylogenetic status within Gentianales, a neighbour-joining (NJ) tree was reconstructed using the complete chloroplast genome sequence of *G. macrophylla* and other 14 species from Gentianales (Figure 1). The result of phylogenetic analysis supported the close relationship of *Gentiana macrophylla* and other 5 species in Gentiana.
